# Synthesis and Reduction Processes of Silver Nanowires in a Silver(I) Sulfamate–Poly (Vinylpyrrolidone) Hydrothermal System

**DOI:** 10.3390/molecules29071558

**Published:** 2024-03-30

**Authors:** Yongling Ying, Rongbo Zheng, Yongjun Zheng, Hongyan Wang, Junfeng Niu, Housheng Xia

**Affiliations:** 1 College of Materials and Chemical Engineering, Southwest Forestry University, Kunming 650224, China; lly2396687411@163.com; 2 College of Biological & Chemical Engineering, Zhejiang University of Science and Technology, Hangzhou 310023, China; 103044@zust.edu.cn (J.N.); 119005@zust.edu.cn (H.X.); 3 School of Marine Science and Technology, Shanwei Institute of Technology, Shanwei 516600, China; 4 Key Laboratory of Bamboo Research of Zhejiang Province, Zhejiang Academy of Forestry, Hangzhou 310023, China; 15990054143@163.com

**Keywords:** silver nanowires, silver(I) sulfamate, PVP end group, decomposition products, reduction process

## Abstract

Silver (Ag) nanowires, as an important one-dimensional (1D) nanomaterial, have garnered wide attention, owing to their applications in electronics, optoelectronics, sensors, and other fields. In this study, an alternative hydrothermal route was developed to synthesize Ag nanowires via modified reduction of Ag^+^. Silver sulfamate plays an important role in the formation of Ag nanowires via controlled release of free Ag^+^. Results of controlled experiments and characterizations such as UV–vis spectroscopy, FTIR, XPS, and ^1^H NMR revealed that sulfamic acid does not function as a reductant, supporting by the generation of free Ag^+^ instead of Ag nanostructures in hydrothermally treated silver sulfamate solution. The initial reduction of Ag^+^ was induced by the combination of poly (vinylpyrrolidone) (PVP) end group and degradation products. This phenomenon was supported by abundant free Ag^+^ in the mixed preheated silver sulfamatic and preheated PVP aqueous solutions, indicating a second and distinct Ag^+^ autocatalytic reduction. Thus, the roles of different reagents and Ag^+^ reduction must be studied for nanomaterial syntheses.

## 1. Introduction

One-dimensional (1D) nanostructures of metals have garnered considerable attention in materials science, owing to their unique electronic, optical, magnetic, and thermal properties, and their use in the fabrication nanoscale electronic, optoelectronic, sensors, and magnetic devices [1,2,3,4,5]. Among such metals, the fabrication of silver (Ag) nanowires is particularly interesting. Bulk Ag has high electrical and thermal conductivities, and has been used in catalysts, optical polarizers, and photonic crystals, and for microbial resistance, wound healing, and biomedical and chemical sensing based on surface-enhanced Raman scattering (SERS) [6,7,8,9,10]. Therefore, Ag nanowires are synthesized using various strategies, such as electrochemical techniques and template-directed (i.e., mesoporous silica and carbon nanotubes) routes [11,12,13,14,15,16,17,18,19,20]. Ag nanowires are synthesized via wet chemical routes [11,15,16,17,18,19,21,22,23,24,25,26]. Ag nanowires can also be synthesized via the orientation of surfactants. Sun and co-workers [16,17,18] synthesized Ag nanowires via a polymer-directed process by reducing Ag nitrate in ethylene glycol using Pt or Ag nanoparticles as the seed with the assistance of poly (vinylpyrrolidone) (PVP). Alternatively, Ag nanowires can be obtained by controlling the concentration of free Ag^+^ in the solution [21,24]. Murphy and co-workers [21] reported on a possible synthesis route for Ag nanowires, i.e., a seedless and surfactant-less wet chemical approach, in which Ag nanowires were prepared by reducing a low-concentration Ag nitrate solution with sodium citrate in the presence of OH^−^. Qian’s research group [24] synthesized uniform Ag nanowires by reducing freshly prepared AgCl with glucose via a hydrothermal process, in which free Ag^+^ was gradually released based on the low solubility of Ag chloride in water [27]. They also successfully prepared Ag/C nanocables using Ag sulfamate to limit the concentration of free Ag^+^ in the solution, such that the rate of nucleation and growth decreased to favor the formation of 1D nanostructure of Ag/C [28]. Rioux et al. [29] demonstrated that Ag nanowires can be obtained by reducing Ag^+^ with PVP hydroxyl end groups in ethylene glycol.

In all cases, reductants such as ethylene glycol, sodium citrate, glucose, salicylic acid, and PVP end groups were exploited to reduce Ag^+^. However, the roles of different reagents were not completely understood. On one hand, sulfamic acid may have functioned as a reductant when Ag sulfamate was used as the Ag precursor. On the other hand, hydroxyl and aldehyde end-groups of PVP caused the initial reduction of Ag^+^. This is because Ag^+^ reduction was substochiometric with respect to PVP end groups, owing to the substantially higher (about 300×) Ag^+^ concentration than the end groups. Thus, whether the decomposition products of PVP caused reduction process or second autocatalytic reduction occurred remained unclear [29,30]. Rioux et al. [29] proposed that PVP end groups induced the initial reduction of Ag^+^ to form Ag^0^_n_ seeds, followed by the autocatalytic reduction of Ag^+^ using ethylene glycol (and not solvent oxidation products) to form Ag nanostructures. However, Ag nanostructures could be generated via simply heating aqueous solutions of PVP and AgNO_3_ in the absence of ethylene glycol [30,31,32,33]. Therefore, elucidating the reduction process of Ag^+^ to form Ag nanowires in a Ag sulfamate–PVP hydrothermal system is challenging.

Herein, an alternative method to synthesize Ag nanowires is proposed using aqueous solutions of PVP and Ag sulfamate [27] as the reductant and Ag precursor, respectively. To investigate the reduction of Ag^+^, preheated Ag sulfamate aqueous solutions and preheated PVP aqueous solutions were allowed to react. Then, NaCl was added to this mixture to test the existence of residual free Ag^+^. On one hand, Ag sulfamate plays an important role in the formation of Ag nanowires via controlled release of free Ag^+^ in the solution. On the other hand, sulfamic acid does not act as a reductant, as it decomposed into SO_4_^2−^ at 120 °C and the initial reduction of Ag^+^ was induced by the combination of PVP end group and PVP decomposition products; subsequently, a second Ag^+^ autocatalytic reduction occurred.

## 2. Results and Discussion

### 2.1. Synthesis of Ag Nanowires

**Scheme 1:**
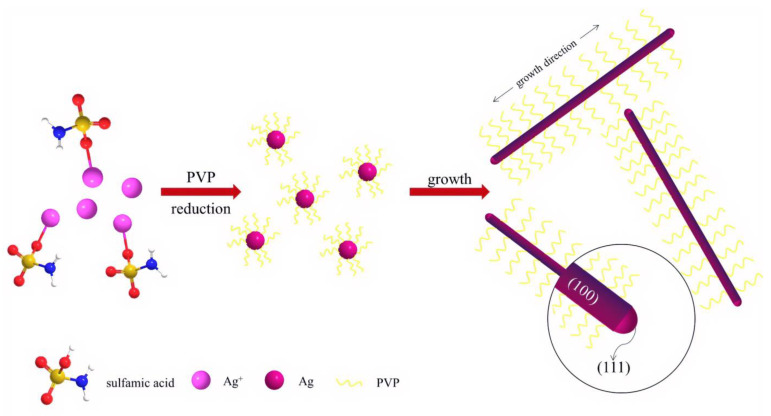
The formation process of Ag nanowires.

As shown in Scheme 1, Ag nitrate first combined with sodium carbonate to form Ag carbonate precipitates. Then, sulfamic acid was immediately added, which reacted with Ag carbonate to form Ag sulfamate. The solution changed from yellow to colorless and transparent, to which PVP was immediately added under stirred magnetically. Ag sulfamate gradually decomposed into Ag^+^ as the temperature increased. Both PVP end groups and PVP decomposition products reacted with Ag^+^ in the solution to form Ag^0^_n_ seeds. PVP bound preferentially with the (111) facet of Ag crystal, guiding the 1D growth along the (111) facet [16,17,18]. Then, a second Ag^+^ autocatalytic reduction occurred. After a long reaction, Ag was generated continuously on the (111) facet and formed Ag nanowires.

### 2.2. Morphology and Phase of Ag Nanowires 

**Figure 1:**
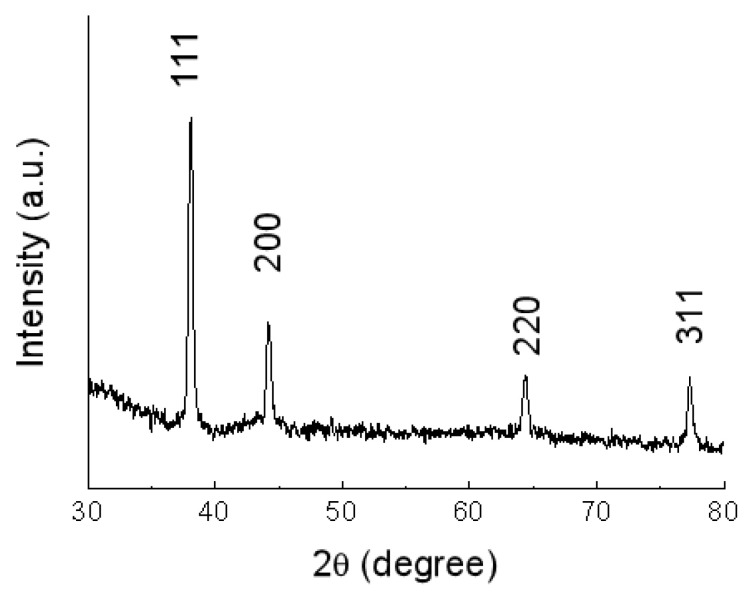
XRD patterns of the Ag nanowires fabricated via hydrothermal synthesis at 180 °C for 48 h.

Figure 1 shows the typical X-ray diffraction (XRD) pattern of Ag nanowires fabricated via hydrothermal synthesis at 180 °C for 48 h [34,35,36]. As shown in Figure 1, all the diffraction peaks can be indexed to face-centered cubic Ag (JCPDS file No. 4-783) [10,34,35,36]. Compared with that of silver porous hollow spheres [34], the ratio of intensities of the peaks (111) to (200) recorded in Figure 1 is larger, might further indicating the 1D growth along (111) direction.

**Figure 2:**
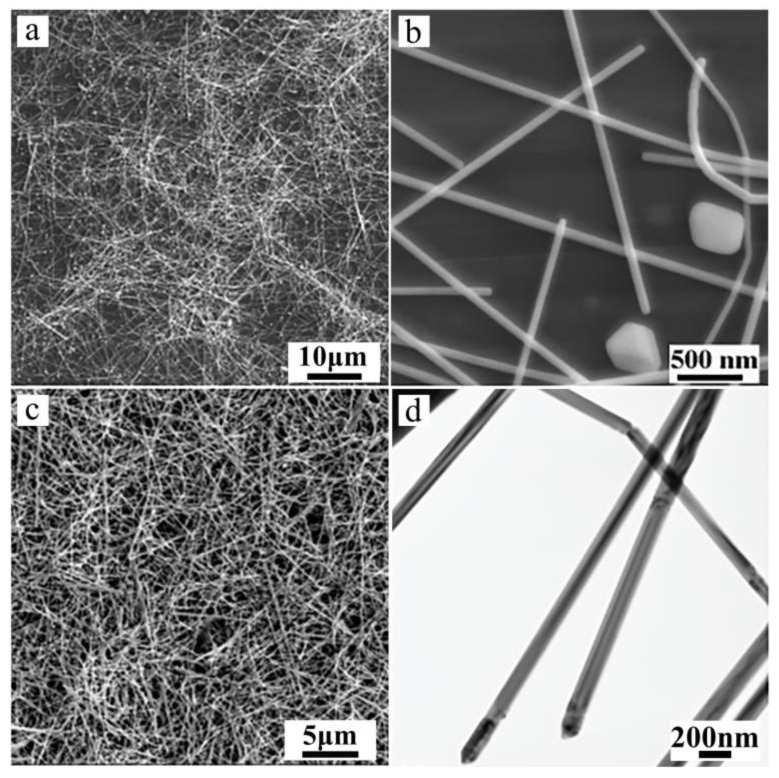
SEM and TEM images of silver nanowires obtained at 180 °C for 48 h: (**a**,**b**) without stepwise centrifugation treatment, (**c**,**d**) after stepwise centrifugation treatment.

Figure 2 shows the scanning electron microscopy (SEM) and transmission electron microscopy (TEM) images [37,38,39,40] of the Ag nanowires [10,25,26]. Figure 2a shows the overall morphology of the Ag nanowires with lengths of up to tens of micrometers. Figure 2b shows the magnified image of the morphology of Ag nanowires, with average diameters of 80–120 nm. A small amount (<10%) of nanoparticles was also found, which can be separated via stepwise centrifugation method [11,15,16,17,18] (Figure 2c,d). Figure 2c shows the typical SEM image of the separated Ag nanowires with diameters of 80–120 nm and lengths up to tens of micrometers. The crystal structures of these Ag nanowires were further investigated via TEM. This result is similar to the Ag nanowires generated via the polyol-directed synthesis in the presence of PVP [15,16,17,18,21].

### 2.3. Formation Process of Ag Nanowires 

**Figure 3:**
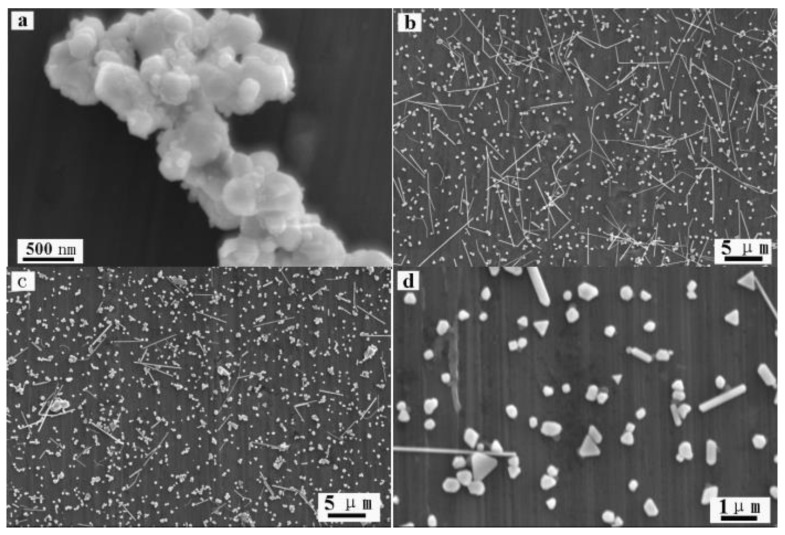
SEM images of Ag nanostructures obtained under different conditions: (**a**) 3 h, (**b**) 12 h, and (**c**,**d**) replacing Ag sulfamate with AgNO_3_ (T = 180 °C, t = 48 h).

**Figure 4:**
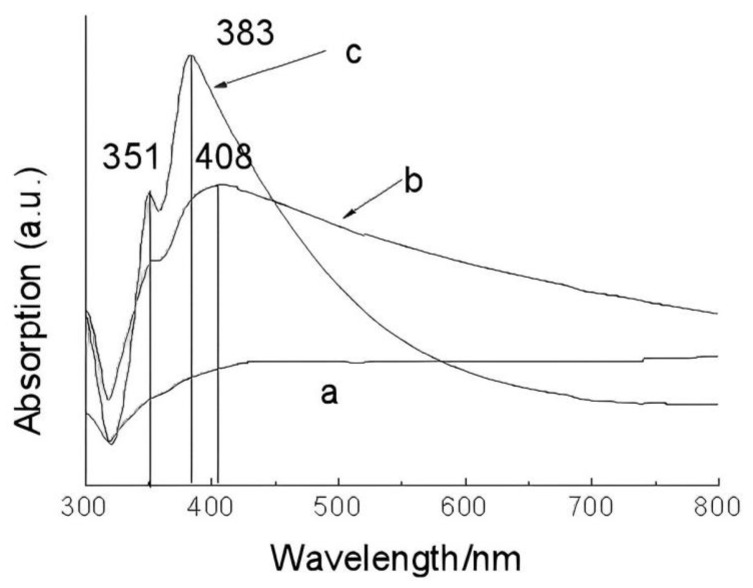
UV–vis extinction spectra of Ag nanostructures hydrothermal treatment for different time: (a) 3 h, (b) 12 h, and (c) 48 h.

The formation of Ag nanowires was investigated by conducting controlled experiments. Results showed that the reaction time and Ag sulfamate affected the formation of Ag nanowires. Figure 3a shows that the nanoparticles with sizes ranging from tens to hundreds nm in the first 3 h of the experiments. As the reaction time was increased to 12 h, some short Ag nanowires appeared along with abundant Ag nanoparticles. The Ag nanoparticles were cubic, triangular, or quasispheroidal (Figure 3b). When the reaction was increased to 48 h, Ag nanowires become predominant (Figure 2). The complex compound, i.e., Ag sulfamate, plays an important role in Ag nanowire formation. As shown in Figure 3c,d, only a small amount of Ag nanorods was generated by replacing Ag sulfonate with AgNO_3_ at 180 °C for 48 h. Ag sulfamate [27] formed by dissolving Ag_2_CO_3_ precipitates in sulfamic acid [28] was used to limit the concentration of free Ag ions (uncomplexed Ag ions) in the solution, such that the rate of nucleation and growth decreased to favor the formation of 1D Ag/C nanostructure [28]. UV–vis spectroscopy was performed to analyze the formation of Ag nanowires [10,41]. Results showed that Ag nanostructures with different morphologies exhibited surface plasmon resonance bands at different frequencies [10,18,34]. Figure 4 shows three typical curves of UV–vis extinction spectroscopy, a very broad absorption of the reaction mixture after 3 h points to a wide range of nanoparticles’ size [18]. As the hydrothermal time increased to 12 h, a shoulder peak appeared at ~351 nm attributed to the plasmon response of Ag nanowires [18,24,42]. A broad peak was also observed at 380–450 nm, which can be attributed to the combined response of Ag nanoparticles and nanowires. The absorption peak observed at ~350 and 390 nm after 48 h can be attributed to the plasmon response of Ag nanowires; this result is similar to those reported previously [10,24,25,26,27].

The formation of Ag nanowires proceeded as follows. First, Ag nanoparticles were generated via the reduction of free Ag^+^ with PVP under hydrothermal conditions. Due to the presence of stable Ag complex, the solution contained very low concentration of free Ag^+^. Thus, the rate of nucleation and growth decreased to favor the formation of 1D Ag nanostructure. Simultaneously, PVP bound preferentially with the (100) facet of Ag crystals, as observed previously [16,17,18]. As the hydrothermal time increased, Ag complex continuously released free Ag^+^ into the solution, which will be reduced by PVP to form Ag atoms. Ag nanowires can be formed via the growth of Ag atoms onto the surface of Ag nucleus to form Ag nanowires with the assistance of PVP.

### 2.4. Reduction Process of Ag Nanowires

Poly (vinyl pyrrolidone) (PVP), a type of polymer comprising a polyvinyl skeleton with polar groups, has been widely used as a capping agent to prevent the agglomeration of Ag nanowires [43,44,45]. As an alcohol with a long alkyl chain, PVP has also been exploited as an ideal reductant to generate noble metal triangular nanoplates via a kinetically controlled process [32,33]. Recently, Rioux et al. [29] obtained Ag nanowires by reducing Ag^+^ with PVP hydroxyl end-groups in ethylene glycol. However, the roles of different reagents were not completely understood therein. On one hand, Ag^+^ reduction is substochiometric with respect to PVP end groups because of substantially higher (about 300×) Ag^+^ concentration than the PVP end groups; therefore, hydroxyl and aldehyde end groups of PVP were used for the initial reduction of Ag^+^ at 140 °C for 26 h to form Ag^0^_n_ seeds of critical sizes [29]. Moreover, PVP decomposition products were also used to induce the reduction of Ag^+^ at 70 °C for 7 h to form Ag nanoparticles [30]. On the other hand, Rioux et al. [29] used PVP end groups to induce the initial reduction of Ag^+^ to form Ag^0^_n_ seeds, followed by the autocatalytic reduction of Ag^+^ by ethylene glycol (and not solvent oxidation products), to form Ag nanostructures. Moreover, Ag nanostructures were generated by simply heating the aqueous solutions of PVP and AgNO_3_ without ethylene glycol [30,31,32,33]. In addition, sulfamic acid may act as a potential reductant when Ag sulfamate was used as Ag precursor. Herein, a series of experiments was conducted to elucidate the reduction of Ag sulfamate in PVP aqueous solutions under hydrothermal conditions.

### 2.5. Sulfamic Acid Does Not Play the Role of the Reductant

**Figure 5:**
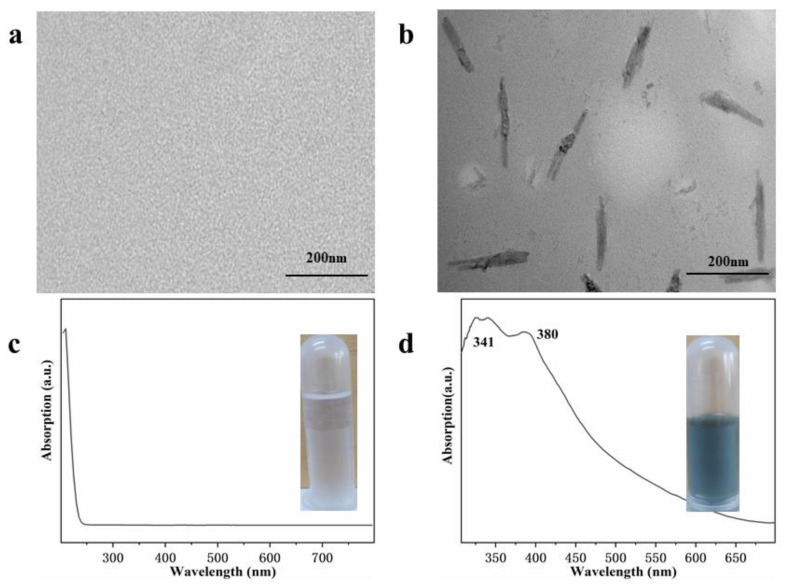
TEM and UV-vis spectra of samples obtained in the absence of PVP (**a**,**c**) and sulfamic acid (**b**,**d**) under same hydrothermal conditions. The inset is the photographs.

To determine the role of sulfamic acid as a potential reductant, a series of controlled experiments were conducted. As shown in Figure 5a,c, Ag nanoparticles were not formed when Ag sulfamate aqueous solution was heated to 180 °C for 48 h. As shown in Figure 5b,d, Ag nanostructures were generated in the absence of sulfamic acid. These results suggest that PVP, rather than sulfamic acid, acts as a reductant for Ag^+^. To further investigate the products obtained from heating Ag sulfamate aqueous solutions, NaCl and Ba(NO_3_)_2_ were added to the Ag sulfamate aqueous solution before and after it was heated to 180 °C for 48 h. The original solution containing NaCl and Ba(NO_3_)_2_ was transparent, indicating that the Ag sulfamate aqueous solution contained very low free Ag^+^ and SO_4_^2−^ concentrations. After heating, the solution containing NaCl and Ba(NO_3_)_2_ turned turbid white, revealing that Ag sulfamate decomposed into free Ag^+^ and SO_4_^2−^ under hydrothermal conditions. Thus, sulfamic acid does not act as a reductant as it decomposes into Ag^+^ and SO_4_^2−^ under hydrothermal conditions.

**Figure 6:**
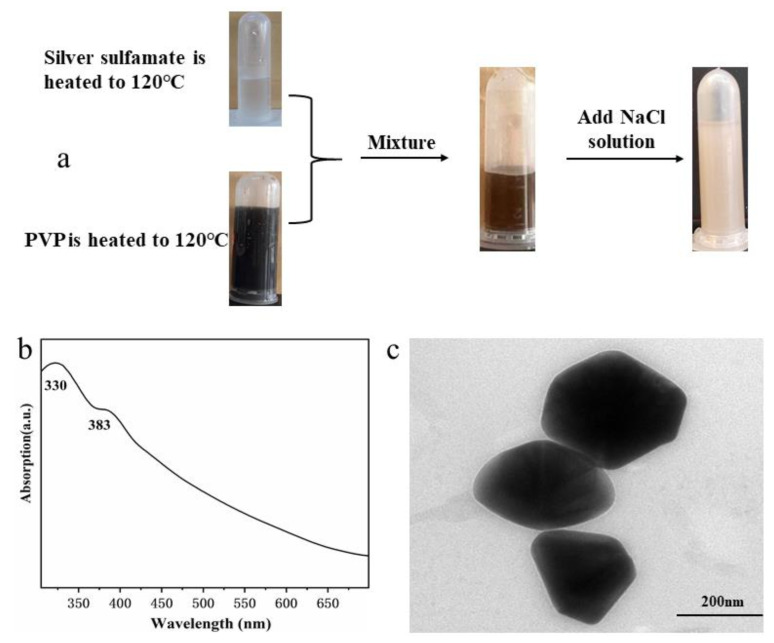
(**a**) Reaction of preheated Ag sulfamate and preheated PVP aqueous solutions, followed by the addition of NaCl, to which yielded large amounts of free Ag^+^. UV-vis spectra (**b**) and TEM image (**c**) of the mixed solutions.

PVP functioned as a reductant in the formation of Ag nanostructures [29,30,31,32,33]. Two possible mechanisms are proposed to explain the reduction process: partial degradation of PVP and PVP end groups. Hoppe et al. [30] proposed that PVP acts as a reductant and undergoes partial degradation, leading to the initial formation of macroradicals. These macroradicals might further cause the metal-accelerated decomposition of low amounts of peroxides present in commercial PVP. Xia and Rioux et al. [29,32,33] demonstrated that the reducing effect of PVP originates from end groups. To investigate the reduction of PVP, a series of experiments was performed. First, Ag sulfamate and PVP aqueous solutions were heated at 120 °C for 12 h, respectively. After heating, the PVP aqueous solution turned dark brown, indicating its partial decomposition [30], whereas Ag sulfamate aqueous solution remained colorless (Figure 6a). These solutions were then mixed at room temperature, revealing the formation of Ag nanoparticles (Figure 6). This trend supports the role of PVP decomposition products as reductants [30]. However, turbid white precipitate was formed after the addition of NaCl into these solutions (Figure 6a), indicating the existence of large amounts of free Ag^+^ (~85%, based on the generated AgCl precipitate). This confirmed that a second reduction process is required to completely reduce Ag^+^ during the formation of Ag nanostructures. Xia and Zhang et al. [30,31,32,33] demonstrated that Ag, Au, Pd, and Pt nanoparticles can be synthesized by promoting the reduction of metal ion and PVP in water at room temperature or 60–80 °C. Rioux [29] assumed that each polymer chain in the commercial PVP has two identical end-group entities, increasing the C_m_ from 147 to 882 mM of 55 K PVP increases the end-group concentration from 0.6 to 3.6 mM. Autocatalytic reduction of Ag^+^ is induced at ~26 h in the presence of 0.6 mM PVP end-groups. According to Rioux’s assumption, the end-group concentration in our hydrothermal system was 0.48 mM and will required more than 26 h to induce the autocatalytic reduction of Ag^+^. However, Ag nanoparticles was formed after hydrothermal treatment at 180 °C for 3 h (Figure 3 and Figure 4). These experimental results and the previous literature indicated that the combination of PVP end group and decomposition products induced the initial reduction of Ag^+^ to form Ag^0^_n_ seeds, followed by a second reduction to completely reduce Ag^+^.

**Figure 7:**
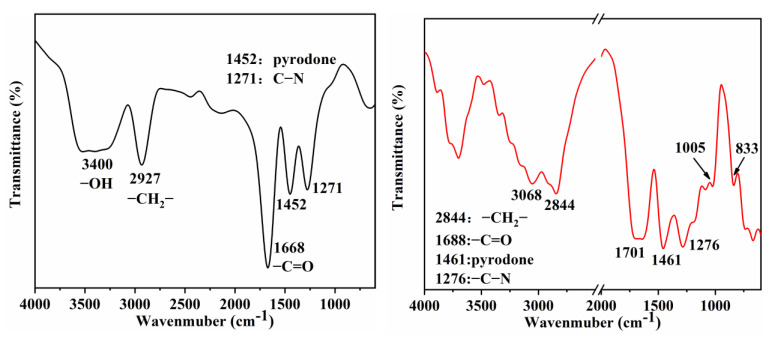
FTIR spectra of PVP obtained from reaction solution before (**left**) and after (**right**) hydrothermal treatment.

We also obtained relevant proof by measuring the FTIR, XPS, and ^1^H NMR of pure and hydrothermally treated PVP. Figure 7 shows the FTIR spectrum of PVP before and after the hydrothermal treatment, wherein a peak can be observed at 3400 cm^−1^, corresponding to the hydroxyl end group. The peak at 2927 cm^−1^ corresponds to the anti-symmetric stretching vibration of methylene in PVP before the hydrothermal treatment. Other peaks at 1668, 1452, and 1271 cm^−1^ correspond to –C=O, pyrodone, and C–N in PVP, respectively. The peak for PVP after the hydrothermal treatment observed at 3400 cm^−1^ disappears and another peak appears at 3051 cm^−1^, corresponding to the O–H stretching vibration in the carboxyl group (dimer). The absorption peak at 1701 cm^−1^ corresponds to the C=O stretching vibration in the carboxyl group, indicating that the hydroxyl end group of PVP is oxidized to carboxyl group during hydrothermal synthesis. Other peaks at 2844, 1461, and 1276 cm^−1^ correspond to the asymmetric stretching vibration, pyrodone, and C–N peaks of methylenes in PVP (Figure 7), respectively, because they remain almost unchanged after the treatment.

**Figure 8:**
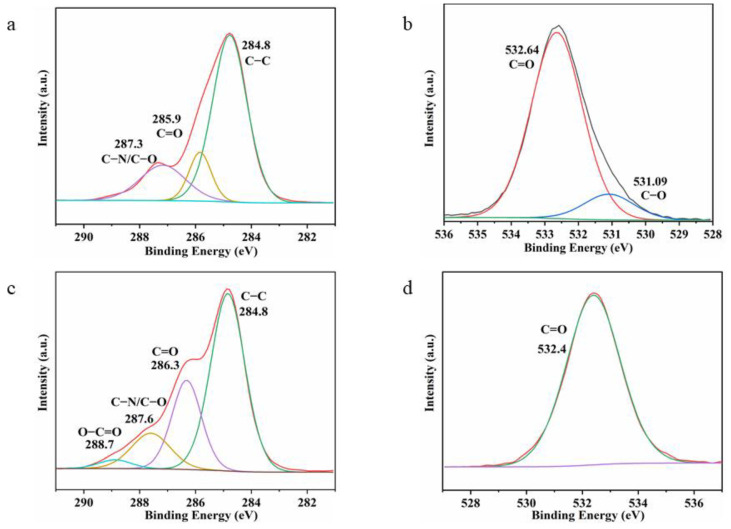
XPS spectra of PVP aqueous solutions before (**a**,**b**) and after (**c**,**d**) hydrothermal treatment.

**Figure 9:**
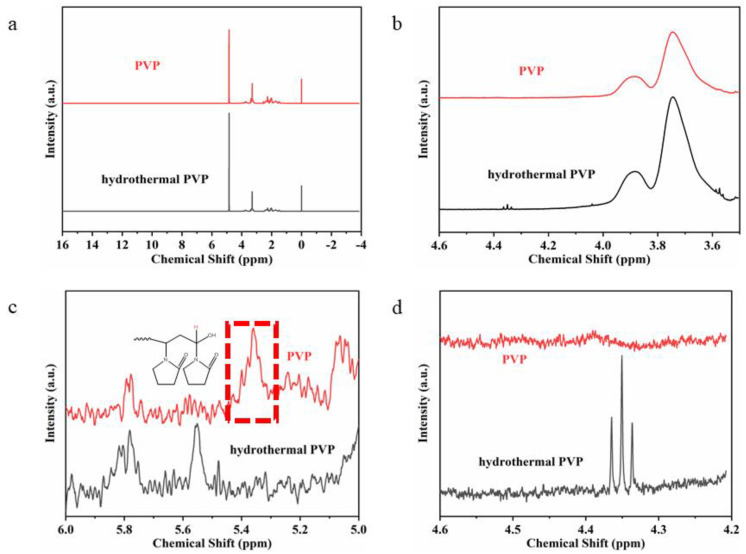
^1^H NMR spectra of pure PVP and reaction product with different magnifications. (**a**) from −4 to 16.; (**b**) from 3.6 to 4.6; (**c**) from 5 to 6; (**d**) from 4.2 to 4.6.

To further determine the role of PVP in the hydrothermal process, the XPS spectra and ^1^H NMR spectra of pure PVP and hydrothermal solutions after the removal of Ag nanowires were compared. Figure 8 shows that both pure and hydrothermally treated PVP showed C–C, C=O, C–N, and C–O peaks. The high-resolution C1s spectra of the hydrothermally treated PVP showed more O–C=O peaks, indicating the oxidation of the hydroxyl end group in pure PVP to carboxyl group in the hydrothermally treated PVP. Moreover, a comparison of the C=O and C–N peak areas revealed that the hydrothermally treated PVP slightly decreased, indicating the conversion of the hydroxyl end group to the carboxyl group. Furthermore, a comparison of their high-resolution O1s spectra indicated the presence of C–O and C=O peaks for pure PVP (Figure 8b) and only the C=O peak for hydrothermally treated PVP. This finding further revealed the oxidation of the end group in PVP (Figure 8d). The ^1^H NMR spectra of pure PVP (Figure 9) shows the presence of –OH end groups [29,30], which disappears in the PVP. In addition, the hydrothermally treated PVP shows three additional peaks between 4.3 and 4.4 ppm, possibly originating from the reaction product [30]. Notably, the peaks of initial –OH end groups and reaction product are weak, indicating a second Ag^+^ autocatalytic reduction process.

As for the second reduction process, Rioux et al. [29] presumed that ethylene glycol functioned as a reductant during the autocatalytic reduction of Ag^+^ because of its high concentration as a solvent. These results indicate that PVP end groups dominate the onset of initial Ag^+^ reduction to form Ag^0^_n_ seeds of critical size, which then initiate autocatalytic Ag^+^ reduction. In the case of the Ag sulfamate-PVP hydrothermal system, we presumed that water functions as a reductant during the autocatalytic reduction of free Ag^+^ based on the Finke–Watzky reduction model [46] as its concentration was higher in the PVP polyol system, similar to ethylene glycol [29].

## 3. Conclusions

Silver nanowires with diameters of ~100 nm and lengths up to tens of micrometers were obtained via the hydrothermal treatment of Ag sulfamate-PVP system. Results showed that Ag sulfamate played an important role in the formation of Ag nanowires via controlled release of free Ag^+^. Moreover, PVP, instead of sulfamic acid, functioned as a reductant, supported by the generation of free Ag^+^ instead of Ag nanostructures in the hydrothermally treated Ag sulfamate solutions without PVP. A post reaction of preheated Ag sulfamate and preheated PVP aqueous solutions was initiated, followed by the addition of NaCl, to confirm the existence of large amounts of free Ag^+^ in the post mixing solutions. This indicated that the initial reduction of Ag^+^ was induced due to the combination of PVP end groups and PVP degradation products, as well as the presence of a second Ag^+^ autocatalytic reduction. We presumed that water functioned as a reductant during the autocatalytic reduction of free Ag^+^ in the Ag sulfamate–PVP hydrothermal system. PVP molecules on the surface of Ag nanowires can be an excellent template to form Ag/SiO_2_ and SiO_2_ nanocables. They can also be used for microbial resistance, wound healing, and other applications. Understanding the roles of different reagents and Ag^+^ reduction would be conducive to the a priori synthesis of nanomaterials.

## 4. Experimental Section

### 4.1. Materials

All chemicals were analytical grade, and were used without further purification. Poly (vinylpyrrolidone) was purchased from Sigma-Aldrich 29k (Lot number MKBJ2815 V, weight average molecular weight, Mw = 30,000), Silver nitrate, sodium carbonate and sulfamic acid were purchased from Beijing Chemical Reagent Factory (Beijing, China). Deionized (DI) water with a resistivity of 18.2 MΩ cm was obtained from a Milli-Q purification system. High temperature reactor purchased from Jinan, Precleaned glass was purchased from Kunming and used without additional cleaning.

### 4.2. Silver Nanowires Synthesis

In a typical procedure, 0.13 g silver nitrate and 0.04 g sodium carbonate were added to 35 mL of distilled water to form a light yellow suspension of silver carbonate under magnetic agitation. Under magnetic stirring, 0.15 g of sulfamic acid was added to the above light yellow suspension to dissolve quickly and gradually become transparent. After stirring for 15 min, 0.5 g PVP (average weight molecular weight, Mw = 30,000) was added and vigorously stirred. The mixture was transferred to a 40 mL Teflon-sealed autoclave and heated at 180 °C for 48 h. After the autoclave was naturally cooled to room temperature, the silver-gray swirling precipitate was obtained by centrifugation and washed three times with distilled water and anhydrous ethanol.

### 4.3. Preparation of Preheated PVP Aqueous Solution

PVP aqueous solution was prepared by adding PVP (1 g) to water (70 mL) to form a transparent solution under magnetic agitation. Then, PVP aqueous solution was transferred to a 40 mL Teflon-sealed autoclave and heated at 120 °C for 12 h. After cooling to room temperature, it was subsequently transferred into a centrifugal tube for further use.

### 4.4. Preparation of Preheated Silver Sulfamate Solution

The silver nitrate (0.39 g) and sodium carbonate (0.12 g) were dissolved in 105 mL of distilled water under magnetic stirring, resulting in the formation of a light yellow suspension of silver carbonate. Subsequently, sulfamic acid (0.45 g) was added to the suspension under continued magnetic agitation for 10 min. Finally, a volume of 35 mL of the silver sulfamate aqueous solution was transferred into 40 mL Teflon-sealed autoclave and heated at 120 °C for 12 h. After the autoclave was naturally cooled to room temperature, preheated silver sulfamate solution was transformed to a centrifugal tube for further use. Similarly, another part of silver sulfamate aqueous solution (35 mL) was also heated at 180 °C for 48 h, followed by stored in a centrifugal tube.

### 4.5. Purification of Silver Nanowires

To purify silver nanowires by step precipitation technique, we firstly centrifuged silver suspensions at 4000 RPM (revolutions per minute) to obtain solid containing nanowires and nanoparticles, then the obtained solid sample was dispersed in water to centrifuge at 3000, 2000, 1000 RPM to obtain solid sample containing nanowires while nanoparticles dispersed in suspension. Subsequently, the silver nanowires were washed three times with distilled water and anhydrous ethanol. Finally, silver nanowires were dispersed in ethanol for further characterization.

### 4.6. Characterization

The obtained products were characterized and analyzed by X-ray diffraction (XRD, Rigaku Dmax2000 with Cu Kα radiation, Rigaku, Tokyo, Japan), the scanning electron microscopy (SEM, XL30ESEM FEG, FEI, USB, Amsterdam, The Netherlands), transmission electron microscopy (TEM, JEOL-2010, Tokyo, Japan), UV–vis-near-infrared (Cary 500 Scan UV-vis-NIR spectrophotometer, Varian, USB, Palo Alto, CA, USA), Fourier transform infrared (FTIR, Bruker, DE, Berlin, Germany) Bruker Model TENSOR27 impact spectrometer. 1H NMR spectra were recorded in CDCl_3_ at 50 °C on a Bruker AMX spectrometer operating at 300 MHz. X-ray photoelectron spectroscopy (XPS, Thermo Scientific, USB, Waltham, MA, USA) analyses was performed on a Physical Electron (PHI, Thermo Scientific, USB) quantum 2000 X-ray photoelectron spectrometer equipped with a monochromatic Alα source (15 kv, 28.8 W) and a hemispherical electron spectrometer equipped with a channel plate and position sensitive detector.

## Data Availability

Data are contained within the article.
